# Esophageal pneumatosis and hematomatosis concomitant with achalasia

**DOI:** 10.1055/a-2329-2150

**Published:** 2024-06-12

**Authors:** Takashi Ueda, Ryuzo Deguchi, Masaya Sano, Hidekazu Suzuki

**Affiliations:** 1 Division of Gastroenterology and Hepatology, Department of Internal Medicine, Tokai University School of Medicine, Isehara, Japan

Although pneumatosis affecting the large intestine, small intestine, and stomach has been documented in the gastrointestinal tract, cases of pneumatosis in the esophagus are infrequent. The patient in this case was an octogenarian man who presented to Tokai University Hospital with the chief complaint of persistent dysphagia. Achalasia was diagnosed using upper gastrointestinal endoscopy and upper gastrointestinal imaging ( Fig. 1 ). Subsequently, hospitalization was deemed necessary due to compromised oral intake.

**Fig. 1:**
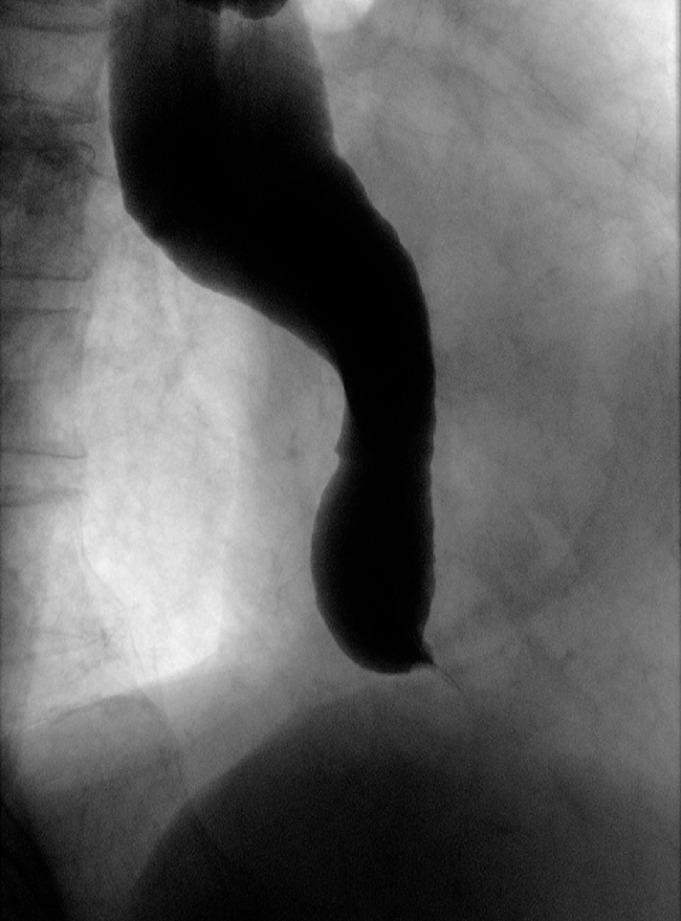
Upper gastrointestinal imaging revealed a smooth stricture, recognized as the "bird beak sign," featuring a maximum esophagus diameter of 3.9 cm and a flexion angle of 130°.

Upon admission, upper gastrointestinal endoscopy revealed conspicuous esophageal bubble lesions and esophageal blood clots ( Fig. 2 ). Conservative treatment measures were taken, and repeat upper gastrointestinal endoscopy was conducted to assess distinctive esophageal findings. Strikingly, all previously identified characteristic esophageal lesions had disappeared ( Fig. 3 ). Testing for viruses, autoimmune diseases, and drugs yielded negative results, and the patient was diagnosed with esophageal emphysema and blood clots caused by achalasia ( Video 1 ).

**Fig. 2:**
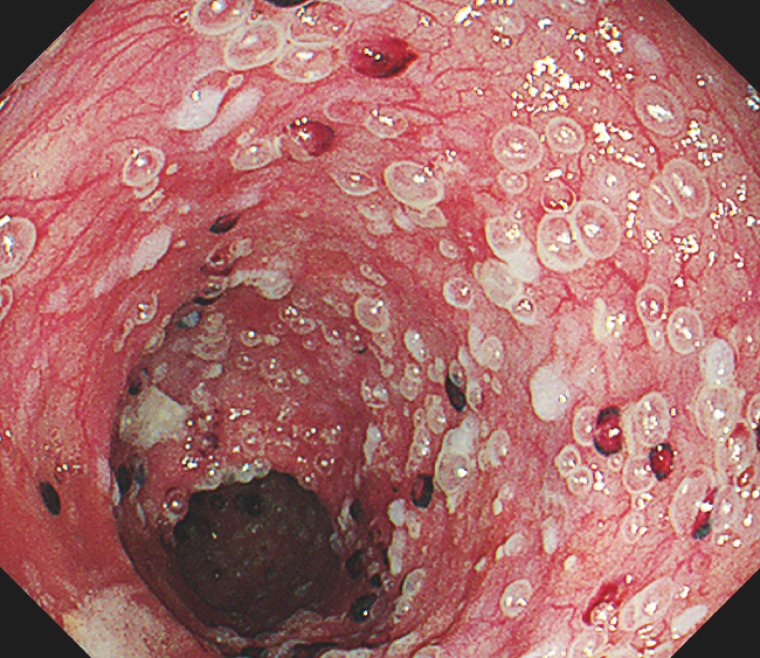
Endoscopy showed esophageal emphysematous changes and the presence of blood clots.

**Fig. 3:**
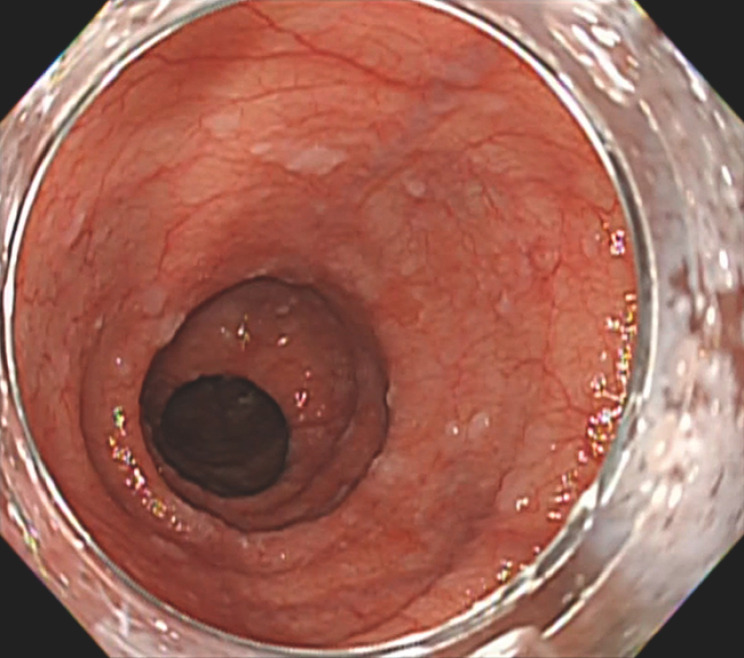
All previously identified characteristic esophageal lesions disappeared.

An unusual case of esophageal pneumatosis and hematomatosis associated with achalasia.Video 1

Gastrointestinal emphysema may manifest as either idiopathic or secondary, with idiopathic cases constituting 15% of all occurrences and secondary cases accounting for the remaining 85% 1 . The etiologies of secondary cases include necrotizing enterocolitis, pyloric stenosis, peptic ulcer disease, jejunoileal bypass, and intestinal obstruction. The mechanism underlying emphysema remains elusive, although the four proposed mechanisms include bacterial involvement, mechanical factors such as increased intraluminal pressure, mucosal damage allowing air entry, and lung disease-related air dislodgment 2 3 4 5 .

In this specific instance, the heightened gastrointestinal lumen pressure resulting from achalasia was postulated to penetrate the gastrointestinal wall through mucosal lacerations and invading small vessels, leading to esophageal mucosal emphysema and blood clot formation. Vomiting, a symptomatic manifestation, is believed to have contributed to esophageal emphysema via the entry of air from the esophagus into mucosal tears induced by mechanical irritation.

Endoscopy_UCTN_Code_CCL_1AB_2AC_3AH

## Notes

Endoscopy E-Videos https://eref.thieme.de/e-videos
*E-Videos* is an open access online section of the journal *Endoscopy* , reporting on interesting cases and new techniques in gastroenterological endoscopy. All papers include a high-quality video and are published with a Creative Commons CC-BY license. Endoscopy E-Videos qualify for HINARI discounts and waivers and eligibility is automatically checked during the submission process. We grant 100% waivers to articles whose corresponding authors are based in Group A countries and 50% waivers to those who are based in Group B countries as classified by Research4Life (see: https://www.research4life.org/access/eligibility/ ). This section has its own submission website at https://mc.manuscriptcentral.com/e-videos .
